# Natural variation in rosette size under salt stress conditions corresponds to developmental differences between Arabidopsis accessions and allelic variation in the *LRR-KISS* gene

**DOI:** 10.1093/jxb/erw015

**Published:** 2016-02-11

**Authors:** Magdalena M. Julkowska, Karlijn Klei, Like Fokkens, Michel A. Haring, M. Eric Schranz, Christa Testerink

**Affiliations:** ^1^Plant Physiology, Swammerdam Institute for Life Sciences, University of Amsterdam, Postbus 94215, 1090GE Amsterdam, the Netherlands; ^2^Department of Phytopathology, University of Amsterdam, Postbus 94215, 1090GE Amsterdam, the Netherlands; ^3^Biosystematics Group, Wageningen University and Research Centre, Wageningen, the Netherlands

**Keywords:** Arabidopsis, development, GWAS, natural variation, rosette size, salt stress.

## Abstract

Natural variation among Arabidopsis accessions is an important genetic resource to identify mechanisms underlying plant development and stress tolerance. To evaluate the natural variation in salinity stress tolerance, two large-scale experiments were performed on two populations consisting of 160 Arabidopsis accessions each. Multiple traits, including projected rosette area, and fresh and dry weight were collected as an estimate for salinity tolerance. Our results reveal a correlation between rosette size under salt stress conditions and developmental differences between the accessions grown in control conditions, suggesting that in general larger plants were more salt tolerant. This correlation was less pronounced when plants were grown under severe salt stress conditions. Subsequent genome wide association study (GWAS) revealed associations with novel candidate genes for salinity tolerance such as *LRR-KISS (At4g08850*), *flowering locus KH-domain containing protein* and a *DUF1639-containing protein*. Accessions with high *LRR-KISS* expression developed larger rosettes under salt stress conditions. Further characterization of allelic variation in candidate genes identified in this study will provide more insight into mechanisms of salt stress tolerance due to enhanced shoot growth.

## Highlight

Study of natural variation of Arabidopsis reveals salinity tolerance to be linked to plant development and expression of novel candidate genes.

## Introduction

*Arabidopsis thaliana* has been used as a model plant in molecular plant biology research for more than 30 years (Somerville and Koornneef, 2002). In the past, mutant screens (Parinov and Sundaresan, 2000) and genome sequencing (Arabidopsis Genome Initiative, 2000) enabled identification and characterization of genes involved in plant development, and biotic and abiotic stress tolerance. Arabidopsis accessions were found to contain many polymorphisms distributed over the entire genome (Clark *et al.*, 2007; Ossowski *et al.*, 2008). The study of development- and stress-related phenotypes of accessions from different geographical regions revealed genetic variation shaped by adaptation to local environment (Beuchat *et al.*, 2010; Fournier-Level *et al.*, 2011; Hancock *et al.*, 2011; Méndez-Vigo *et al.*, 2011). Factors such as temperature, rainfall and day length were suggested to be responsible for local adaptation.

Salt stress is one of the major abiotic stresses, causing a decline in areas suitable for agriculture. Salt stress exposure results in immediate activation of non-specific cation channels (NSCCs), generation of signals such as phospholipids, reactive oxygen species (ROS) and Ca^2+^ waves, and protein kinase activation, leading to induction of hormone signalling, stomatal closure and plant growth alterations (Duan *et al.*, 2013; Galvan-Ampudia *et al.*, 2013; Geng *et al.*, 2013; Jiang *et al.*, 2013; Choi *et al.*, 2014; Julkowska *et al.*, 2014a; Julkowska *et al.*, 2014b; Julkowska and Testerink, 2015). These quick responses are thought to be independent of sodium ion accumulation in shoot tissue (Roy *et al.*, 2014). In the later stages of salt stress exposure, growth is mainly impaired by sodium accumulation in shoot tissue, membrane dysfunction and oxidative stress, leading to tissue damage and premature leaf senescence (Munns and Tester, 2008). Compartmentalization of sodium ions into the vacuoles and non-photosynthetic tissues is the main mechanism regulating ion homeostasis and enhancing salt stress tolerance (Roy *et al.*, 2014). Screens of Arabidopsis accessions in Na^+^ shoot accumulation provided evidence that allelic variation present in *HKT1* and *CIPK16* plays a role in cellular and intracellular compartmentalization of sodium ions, respectively (Baxter *et al.*, 2010; Roy *et al.*, 2013).

Natural variation in growth recovery in the later stages of salinity stress depends in part on ion exclusion from the shoot and efficient ion compartmentalization at cellular and intracellular levels (Rus *et al.*, 2006; Moller *et al.*, 2009; Munns *et al.*, 2012; Roy *et al.*, 2013). However, the allelic variation underlying maintenance of rosette growth, rather than ion exclusion, is yet to be explored. Although maintenance of growth under salt stress condition is widely used as an index for salt stress tolerance (Roy *et al.*, 2014), the relationship between plant size and salt stress tolerance is not clear. Slower growth is thought to enhance salt tolerance due to relatively low transpiration rate and reduced sodium loading to the xylem vessels. On the other hand, enhanced growth results in larger plant volume, which reduces sodium concentration and delays onset of premature leaf senescence (Hasegawa, 2013).

Here we focus on the effect of salinity on rosette size in plants grown for 2 weeks in control or severe salt stress conditions. We observed strong correlation between rosette size in control and 300mM NaCl, which suggests that natural variation in salt stress tolerance depends to a large extent on developmental differences between Arabidopsis accessions. Subsequent genome wide association study (GWAS) revealed no overlap in associated loci between control and salt stress conditions for any of the traits studied. One of the identified candidate genes associated with dry weight (DW) at 500mM NaCl was a *Leucine-Rich Repeat Kinase family protein Induced by Salt Stress* (*LRR-KISS*). Furthermore, we identified six loci associated with fresh weight (FW) under salt stress conditions. Another five loci were identified associated with the ratio between FW and projected rosette area (FWpPRA) in salt stress conditions, which was used as a proxy for water retention in the shoot tissue. Further study of T-DNA insertion lines and accessions showing natural variation in expression levels of KH-domain and DUF1639 containing proteins, associated with FW and FWpPRA, respectively, confirmed the identified genes to be involved in rosette development in control and salt stress conditions. Analysis of accessions varying in *LRR-KISS* expression revealed that high transcription of *LRR-KISS* correlated with high rosette size under saline conditions. This study shows the use of simple and inexpensive phenotyping methods for screening natural variation, leading to identification of salt stress sensitive and tolerant accessions and novel candidate genes involved in rosette development during salt stress.

## Materials and methods

### Plant growth conditions

We selected 160 accessions from the HapMap population based on data available on the sodium/potassium accumulation in leaf tissue (Baxter *et al.*, 2010) and survival data (Katori *et al.*, 2010). Two populations containing a different selection of 160 different accessions were used in the experiments conducted in the greenhouse of the University of Amsterdam during the period between September and October of 2012 and 2013, with a controlled regime of 16h light and a temperature of 21 °C. The populations of Arabidopsis accessions differed between the two experiments, with 81 overlapping accessions. The accessions used are listed in Supplementary Tables S1 and S2 at *JXB* online. The seeds were stratified for 3 days at 4 °C in 0.1% agar to ensure equal exposure to cold treatment and to synchronize germination under greenhouse conditions. Accessions were distributed over the trays containing 5×8 pots (Verspeentrays Deens format, Desch-plantpak, The Netherlands), each with volume of 127ml, filled with 150g soil (Zaaigrond nr 1, SIR 27010-15, Jongkind BV, The Netherlands) following a random design. No additional nutrients were supplemented. Twenty days after germination, eight (in year 2012) and 12 (in year 2013) representative seedlings per accession were selected and treated with 1L of rainwater, and 500 or 300mM NaCl from below (experiments conducted in 2012 or 2013, respectively). The treatment was repeated every second day for 15 days by replacing liquid remaining in a tray with 1L of treatment solution, with no additional watering from the top. The concentration of 500mM NaCl applied in the first experiment was chosen based on the work published by Katori *et al.* (2010), and was lethal to some of the accessions (dead plants were included in the analysis). In order to score more subtle phenotypes, in the experiment conducted in 2013, plants were treated with 300mM NaCl.

The number of biological replicas varied for the individual traits. In the first experiment conducted in 2012 all data were collected from four plants per condition, while in the experiment conducted in 2013 fresh and dry weight and electrolyte leakage data were measured for three biological replicates and projected rosette area from six biological replicates.

### Projected rosette area measurements

The rosette-projected area was determined by photographing the rosettes of 35-day-old plants against a blue background. The pictures were taken in the flash-cross polarization setting to reduce the light reflection. The images were cropped with Adobe Bridge software and the scale was determined based on the size of the label photographed with individual rosettes. The surface of the rosette was determined an ImageJ script (see batch final.doc file) as a percentage of the surface of the scaled picture and recalculated into square centimetres. Data collected from a single replicate per accession or extreme outliers within accessions were removed from subsequent analysis.

### Fresh and dry weight measurements

In the experiment conducted in 2012, the fresh and dry weight of the rosette and flowering stem were measured. In the experiment conducted in 2013, the flowering stem was excluded from fresh and dry weight measurements, to specifically assess rosette growth. The fresh and dry weights were determined with the accuracy of 0.001g using regular lab scales. The dry weight of the aboveground tissue was measured after drying the material for 48h at 60 °C. The water mass of individual plants was calculated by subtracting dry weight from fresh weight, while water content was calculated by dividing water mass by dry weight. The data were examined for outliers within individual accessions, which were removed from subsequent analysis.

### Electrolyte leakage

Three leaf discs with a diameter of 1cm were excised from the three largest leaves of 35-day-old plants. The leaf discs were washed in 50ml of MQ water for 30min on an orbital shaker under a TL lamp. Subsequently, each leaf disc was transferred to 1ml of 0.01% Silwet solution in MQ water and vacuum infiltrated for 2×2min. The samples were then incubated on an orbital shaker under TL light for 1h. The electrolyte leakage was determined by measuring the conductivity of the Silwet solution after 1h of incubation on an orbital shaker. The total electrolyte leakage was measured after boiling the samples. The conductivity was measured with a Horiba B-173 twin conductivity meter calibrated with the supplied solution. The relative electrolyte leakage as a measure of cell damage was calculated by dividing the electrolyte leakage of fresh samples by the conductivity measured after boiling the leaf discs. Three plants were used for the biological replicates per accession per condition. The data were examined for outliers, which were removed from subsequent analysis.

### Candidate gene identification by means of GWAS

The phenotypic data collected were combined with the published genomic data on accessions from a 250k single nucleotide polymorphism (SNP) chip array with average SNP density of one SNP in 500bp (Atwell *et al.*, 2010). We performed independent GWAS using the entire SNP data set. The associations between each SNP and individual root system architecture (RSA) phenotypes were tested using a scan_GLS algorithm (Kruijer *et al.*, 2015) based on EMMA-X. The method implements two corrections for multiple testing in order to minimize the false discovery rates. In the first, more strict method, the LOD threshold is determined by –log10(α×*P*-value^−1^), where α is significance level of either 0.01 or 0.05. The other method uses the number of markers (*p*) replaced by the number of effective tests approach, as in Gao (2011). Both of the methods were applied to individual phenotypic values per accession. The selection of candidate loci was performed based on the minor allele frequency, LOD score and number of significantly associated SNPs within one locus (within a 10kb interval from each other) and the trait heritability (Table 2). The putative candidate loci are listed in Table 3.

### Role of putative candidates in salt stress tolerance

In order to examine the role of putative candidate genes in salt stress tolerance, T-DNA insertion lines of genes underlying the loci identified with GWAS were examined for their phenotype in salt stress tolerance. The T-DNA insertion lines and primers used for genotyping are listed in Supplementary Table S5. The seeds of T-DNA insertion lines and Col-0 were germinated under short-day conditions (21 °C, 70% humidity, 10/14h light/dark cycle). One week after germination the plants were treated with 0 or 75mM NaCl applied from above. The treatment was repeated every other day. After 6 weeks the plants were harvested and fresh weight of the rosettes was measured. Fifteen replicas per genotype per condition were used. The data were examined for outliers, which were removed from subsequent analysis. The statistical analysis was performed in Excel and SPSS was used for one-way ANOVA with Tuckey’s *post hoc* test for significance. The natural variation and response to salt in expression of candidate genes was studied using the eFP browser (Lempe *et al.*, 2005; Kilian *et al.*, 2007).

### Sequence similarity analysis of the selected loci

Sequence information of 160 accessions of which genome sequence is available (Supplementary Table S6) and which belong to the HapMap population was downloaded from the 1001 Genomes Project website (1001genomes.org) and aligned with ClustalO. The sequences were compared for sequence similarity, gaps and missing data and plotted using Gnu-plot software package.

## Results

### Natural variation in rosette-related phenotypes under salt stress

**Fig. 1.**
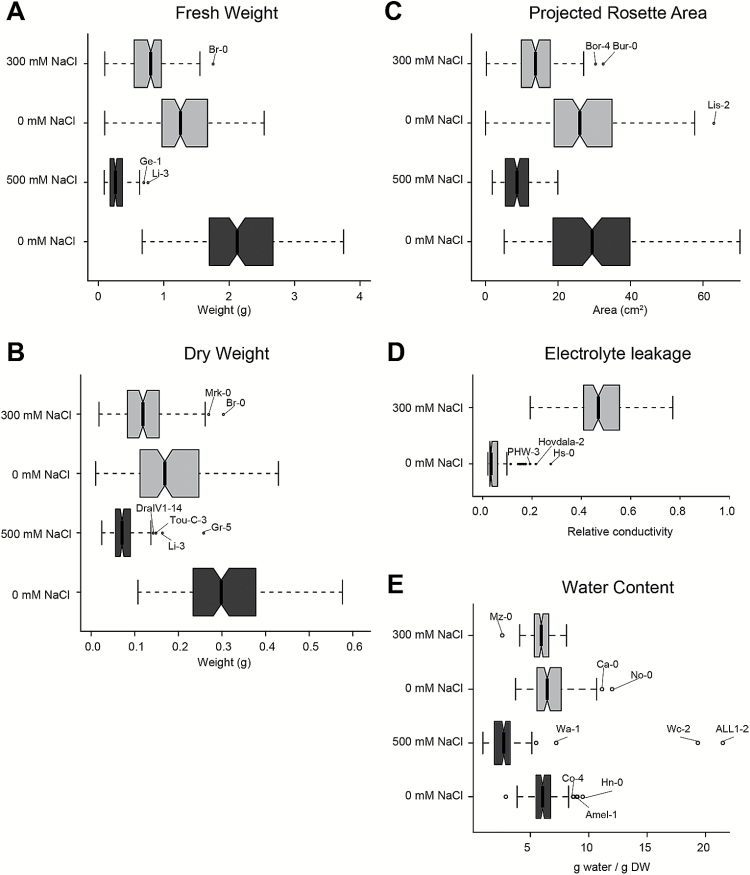
Natural variation in salinity tolerance observed for Arabidopsis accessions. The phenotypes of plants grown in control and salt stress conditions were studied in order to examine the natural variation within the population studied. The average value per accession was calculated. (A) fresh weight (FW) and (B) dry weight (DW) of the entire above-ground part were determined in the experiment conducted in the year 2012 (dark box plots), while fresh weight of the rosette was measured in the experiment conducted in the year 2013 (light box plots). (C) Projected rosette area (PRA) was significantly smaller for the plants watered with 300 and 500mM NaCl when compared with plants grown in control conditions. (D) Electrolyte leakage (EL) increased for plants watered with 300mM NaCl compared with plants grown in control condition. (E) Water content calculated as (FW–DW)/DW was significantly decreased in both salt stress treatments relative to control conditions. The boxplots represent the median length as observed in 160 accessions for FW, DW, PRA and WC and in 113 accessions for EL. The whiskers extend to data points that are less than 1.5× the interquartile range (IQR) away from the first and third quartile. Notches represent 1.58×IQR/√*n* and give 95% confidence that two medians differ. Accessions representing outliers for a trait are indicated.

To determine natural variation in shoot growth under salt stress conditions, Arabidopsis accessions were screened for their rosette size in control and saline conditions. Two large-scale experiments, each consisting of 160 Arabidopsis accessions selected from the HapMap population, were conducted in greenhouse conditions using rainwater containing 0, 300 or 500mM NaCl starting from 20 days after germination (Supplementary Fig. S1). The treatment was repeated every second day for 15 days, after which projected rosette area (PRA), fresh weight (FW) and dry weight (DW) were determined. In the experiment using 500mM NaCl, fresh and dry weight of rosette and flowering stem were measured (Supplementary Fig. S1A and Fig. 1A–C). In the experiment using 300mM NaCl as salt stress treatment, the flowering stem was excluded from the measurements. Additionally, in this experiment the relative electrolyte leakage (EL) was determined for 113 accessions (Supplementary Fig. S1B and Fig. 1D). The selection of accessions used for the individual experiments is listed in Supplementary Tables S1 and S2. The populations of Arabidopsis accessions differed between the two experiments, with 81 overlapping accessions. The projected rosette area was highly correlated between the experiments for plants grown in control and salt stress conditions, while the biomass could not be compared since different tissues were harvested for the individual experiments (Supplementary Figs S1 and S2). High correlations in projected rosette area between the experiments indicate high reproducibility of the phenotypes measured.

**Fig. 2.**
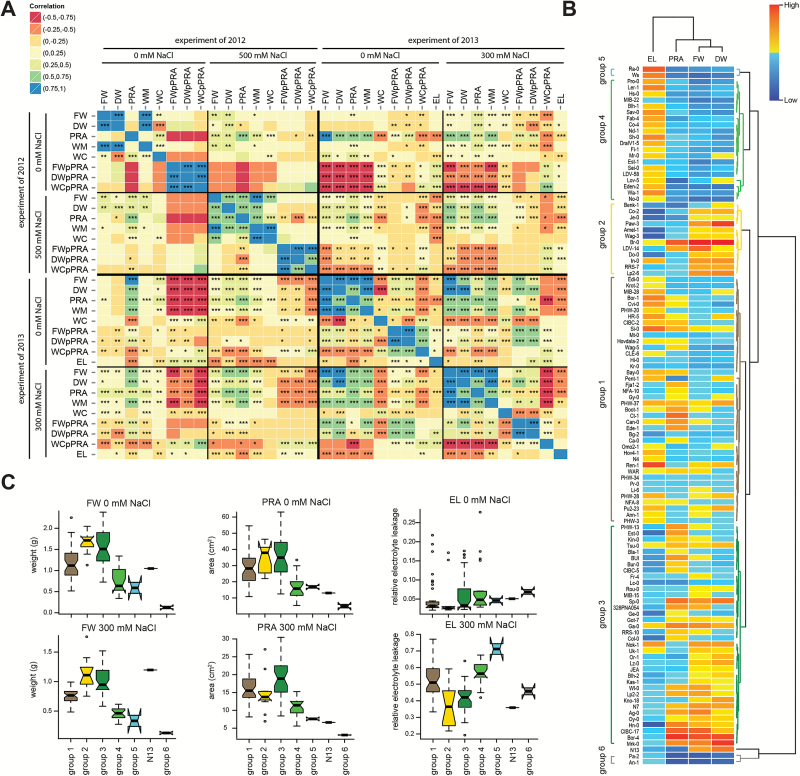
Natural variation reveals a relationship between development and salt stress tolerance. (A) The correlation between phenotypic traits was determined by calculating the Pearson correlation coefficients (*r*). The correlation heat map represents the *r*-values, with orange squares representing negative and blue positive correlations. The significance of *r*-values is indicated with *, ** or *** for levels of 0.05, 0.01 and 0.001, respectively. The correlation analysis was performed on 64 accessions included in both experiments and of which EL was measured. The *r*-values are listed in Supplementary Tables S3 and S4. (B) Clustering of the rosette phenotypes of plants grown in 300mM NaCl was performed using the Ward linkage method. The variation in the phenotypes is represented in different colours normalized with *z*-Fisher transformation. (C) The fresh weight (FW), projected rosette area (PRA) and relative electrolyte leakage (EL) phenotypes in control and salt stress conditions was observed to differ between identified clusters of accessions. The boxplots represent the median phenotype of all accessions belonging to the specific cluster. The whiskers extend to data points that are less than 1.5× the interquartile range (IQR) away from the first and third quartile. Notches represent 1.58×IQR/√*n* and give 95% confidence that two medians differ.

While high biomass and rosette projected area would indicate salinity tolerance, increased electrolyte leakage corresponds to cell damage and can be used for indicating salt stress sensitive accessions. Considerable natural variation was observed for all phenotypes studied (Fig. 1). Both salt stress treatments significantly decreased the rosette area and weight when compared with control conditions (Fig. 1A–C). The relative electrolyte leakage increased approximately 10-fold in leaf discs excised from salt-treated plants (Fig. 1D). Moreover, plants grown under saline conditions showed reduced variability in the rosette size. This reduction in phenotype distribution was more severe for plants growing at 500mM NaCl than at 300mM NaCl (Fig. 1A–C). On the other hand, variation in the relative electrolyte leakage increased after salt stress treatment (Fig. 1D). Interestingly, the accessions identified as outliers differed among different traits and conditions studied (Fig. 1 and Supplementary Fig. S3). In order to establish intrinsic correlations between quantified rosette traits, Pearson correlation coefficients (*r*) were calculated (Fig. 2A, Supplementary Fig. S4 and Supplementary Tables S3 and S4). Fresh and dry weights were observed to be in strong correlation in all conditions studied (*r*>0.72), while the correlation between the rosette area and weight was weaker yet significant (*r*>0.65). Water content showed strong (*r*=0.71) correlation with fresh weight only in plants treated with 500mM NaCl, while for plants grown in control conditions the correlation was either negative (both experiments) or non-significant (300mM NaCl). Electrolyte leakage was in negative correlation with rosette size only under salt stress conditions. Although significant correlations between different phenotypes were observed, varying correlation strength as well as identification of unique outliers suggests the presence of different strategies among Arabidopsis accessions in their response to salt stress.

Arabidopsis accessions differed in rosette size measured in control conditions. Those developmental differences between individual accessions could explain their respective performance under salt stress conditions. To investigate the relationship between the rosette size in control and salt stress conditions, accession-specific correlations of traits between control and salt stress conditions were examined (Table 1). Rosette weight and area were significantly correlated between control and salt stress treatments in both experiments performed, albeit the correlations were stronger when plants were treated with 300mM NaCl (Supplementary Tables S3 and S4). Interestingly, the correlation between water content in salt and control condition was significant only when plants were treated with 300mM NaCl. Those observations indicate that the differences in response to lower salt concentrations are linked to developmental phenotypes observed in control conditions. The weaker correlations observed between control plants and plants watered with 500mM NaCl suggest that mechanisms other than development play an important role in maintenance of rosette growth in severe salt stress conditions.

**Table 1.** Overview of phenotypic traits collected The effect of salt stress on individual rosette parameters was studied by comparing the value observed in salt stress with control conditions.

**Trait**	**Year**	**[NaCl] (mM**)	**Correlation coefficient (Control vs Salt**)
Fresh weight (g)	2012	500	0.26*
	2013	300	0.79***
Dry weight (g)	2012	500	0.28*
	2013	300	0.77***
Projected rosette area (cm^2^)	2012	500	0.66***
	2013	300	0.63***
Water mass^a^ (g)	2012	500	0.24
	2013	300	0.64***
Water content^b^	2012	500	0.21
	2013	300	0.33**
Fresh weight per projected rosette area (g cm^–2^)	2012	500	0.2
	2013	300	0.53***
Dry weight per projected rosette area (g cm^–2^)	2012	500	0.15
	2013	300	0.62***
Water content per projected rosette area (cm^–2^)	2012	500	0.54***
	2013	300	0.48***
Electrolyte leakage (%)	2013	300	0.07

The significant correlations between salt stress and control conditions are indicated with * or ** for significance levels of 0.05 and 0.01, respectively.

^a^ Water mass=fresh weight–dry weight.

^b^ Water content=water mass/dry weight.

To identify patterns in rosette size-related phenotypes, the phenotypes of 113 accessions treated with 300mM NaCl, of which all the collected phenotypes were available, were clustered based on the rosette weight, area and electrolyte leakage measured in salt stress conditions (Fig. 2B). The clustering revealed seven distinct groups. Three groups (5, 6 and N13) were represented by one or two accessions. The majority of the accessions belonged to group 1, which showed intermediate rosette weight and area and relatively high electrolyte leakage (Fig. 2C). Accessions with the largest rosette sizes in control and salt stress conditions belonged to group 2 and 3, which both showed relatively low electrolyte leakage under saline stress conditions. Among the accessions belonging to group 3 we identified Tsu-0 and Bur-0, previously established as accessions with enhanced salinity tolerance (Rus *et al.*, 2006; Katori *et al.*, 2010). Accessions in group 4 showed small rosette size in both control and salt stress conditions and high electrolyte leakage in salt stress conditions. The patterns in rosette size and cell damage observed for the majority of accessions reveal a general trend that plants with large rosettes suffer less from salt stress-induced cell damage. Only two accessions belonging to group 6 (Pa-2 and An-1) showed relatively low electrolyte leakage despite small rosette size under salt stress conditions. The diversity of collected phenotypic measurements allowed identification of distinct groups of accessions with general patterns of salt response correlation with rosette size as well as identification of accessions that do not follow the general trend.

### Identification of putative candidate genes through GWAS

The phenotypic data collected from control and saline conditions were used as an input for genome wide association study (GWAS) (Kruijer *et al.*, 2015). The heritability of individual traits (Table 2) varied between 0.156 for projected rosette area at 300mM NaCl and 0.912 for electrolyte leakage in control conditions. Phenotypes with low heritability (<0.2) were excluded from further analysis. Candidate loci were selected based on the minor allele frequency, LOD score and number of significantly associated SNPs within one locus. In total 15 unique associations were identified (Table 3). Fresh weight of plants treated with 500mM NaCl was associated with six genomic regions, and three neighbouring significant SNPs with allele frequency above 0.03 were found on chromosome 3. Dry weight of severely stressed plants was associated with only one SNP on chromosome 4, with low allele frequency (0.01). No significant associations were observed for the fresh weight, dry weight or projected rosette area of plants treated with 300mM NaCl. The ratio of fresh weight per projected rosette area (FWpPRA) in salt (300mM NaCl) conditions, used as a proxy for water retention, was associated with five loci. No overlap in associations was observed, either between salt stress and control conditions or for different phenotypes. The phenotypes collected in control conditions in the experiment performed in the year 2012 did not yield any significant associations, while the associations found for phenotypes scored under control conditions in experiment performed in 2013 are listed in Table 3.

**Table 2.** Trait heritability The heritability for individual rosette phenotypes was calculated on individual values.

**Trait**	**0mM NaCl**	**300mM NaCl**	**500mM NaCl**
Fresh weight	0.3932	—	0.6215
Dry weight	0.5204	—	0.3975
Projected rosette area	0.6674	—	0.3703
Fresh weight	0.7246	0.0654	—
Dry weight	0.5991	0.6584	—
Projected rosette area	0.2170	0.1569	—
Electrolyte leakage	0.9120	0.3923	—
Fresh weight per projected rosette area	0.5889	0.5325	—
Dry weight per projected rosette area	0.5805	0.5214	—

**Table 3.** Candidate loci associated with salinity tolerance identified with GWAS The putative candidate genes were selected based on the LOD score, minor allele frequency of the SNP associated with the rosette size phenotype and number of SNPs associated with single locus.

Rosette phenotype	Chr	Position	LOD	Col-0 allele freq.	Candidate gene	Description of gene directly underlying identified SNP
Dry weight, 500mM NaCl	4	5641647	7.81	0.99	AT4G08850	Leucine-rich repeat receptor-like protein kinase family protein
Fresh weight, 500mM NaCl	2	11102323	7.48	0.99	AT2G26060	CYTOSOLIC IRON-SULFUR PROTEIN ASSEMBLY 1
	2	19219192	8.38	0.99	AT2G46760	D-Arabinono-1,4-lactone oxidase family protein
	3	1168187	9.01	0.97	AT3G04400	Embryo defective 2171 (EMB2171)
	3	1244780	12.59	0.97	AT3G04600	Nucleotidylyl transferase superfamily protein;
		1246615	7.91	0.92	AT3G04605	Member of a domesticated transposable element gene family (MUSTANG1)
		1253713	8.85	0.96	AT3G04610	Flowering locus KH domain (FLK)
	3	2181206	5.41	0.97	AT3G06920	Tetratricopeptide repeat (TPR)-like superfamily protein
		2184962	7.51	0.98		
	4	8688200	8.97	0.01	AT4G15233	ABC-2 and Plant PDR ABC-type transporter family protein
		8704079	4.46	0.03	AT4G15240	Protein of unknown function (DUF604)
Fresh weight per projected rosette area, 300mM NaCl	1	8898857	7.58	0.98	AT1G25370	Unknown
	1	19111255	10.78	0.99	AT1G51530	RNA-binding (RRM/RBD/RNP motifs) family protein
	5	200900	7.9	0.02	AT5G01500	Encodes an ATP/ADP carrier that is located to the thylakoid membrane involved in providing ATP during thylakoid biogenesis and turnover
	5	21253671	7.79	0.98	AT5G52340	A member of EXO70 gene family, putative exocyst subunits, conserved in land plants
		21253918	7.79	0.98		
	5	25260662	7.37	0.98	AT5G62940	HIGH CAMBIAL ACTIVITY2 (HCA2) - induces the formation of interfascicular cambium and regulates vascular tissue development in the aerial parts of the plant.

Chr: chromosome.

**Fig. 3.**
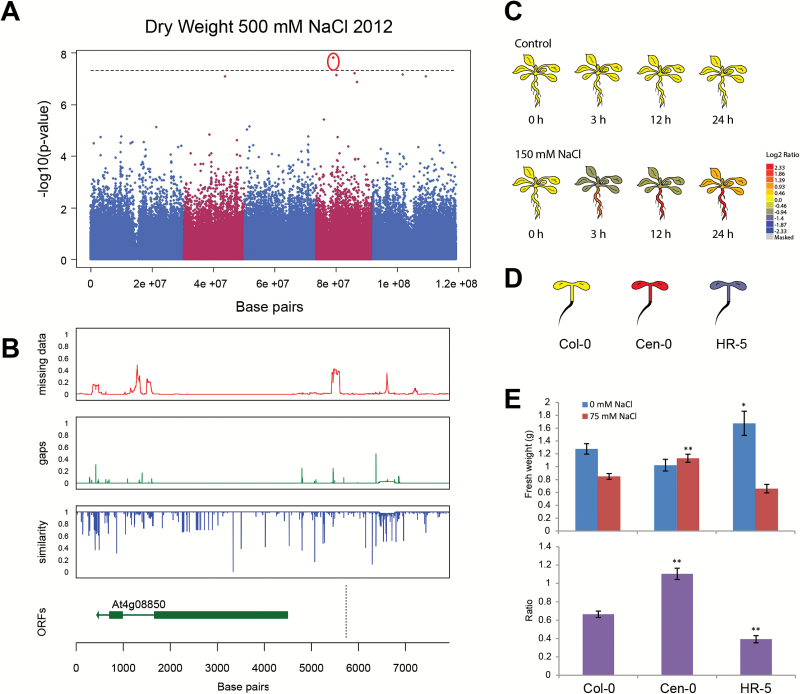
Natural variation in dry weight associates with *LRR-KISS*. (A) Genome wide association study performed on the dry weight of plants watered with 500mM NaCl with minor allele frequency of 0.01 showed an association with an SNP on chromosome 4. The identified SNP is circled in red. The association was not observed for any other trait or condition studied. (B) The SNP was located in the promoter region of At4g08850, encoding a leucine-rich repeat kinase-like protein. The natural variation in the promoter and gene-coding region was studied in 162 accessions sequenced by the 1001 Genomes Project belonging to the HapMap population (Supplementary Table S6). The red graph represents the portion of missing data, while the green graph represents deletions present in accessions other than Col-0. The blue graph represents the sequence similarity compared to Col-0. The open reading frames (ORFs) are represented in the lowest graph. The location of the SNP associated with dry weight at 500mM NaCl is represented with the dashed line. (C) The expression of At4g08850 is induced in the root after exposure to salt stress relative to expression in control conditions (Kilian *et al.*, 2007) as reported by the eFP browser. (D) Natural variation in *LRR-KISS* (At4g08850) expression is enhanced in Cen-0 and reduced in HR-5 accessions relative to Col-0 (Lempe *et al.*, 2005) as reported by the eFP browser. (E) The natural over-expression line Cen-0 and knock-down line HR-5 of *LRR-KISS* were tested for rosette growth in control and salt stress conditions. One-week-old plants grown under short-day conditions were treated with 0 or 75mM NaCl applied from above for 6 weeks. The fresh weight of the rosette was used as an indicator for growth and salinity tolerance. The bars represent the average fresh weight of the rosette as calculated from 15 biological replicates per line per conditions. The error bars represent standard error. The phenotypes of both accessions were tested for significant differences from Col-0, which were calculated using ANOVA with Tukey’s *post hoc* test. The significance is indicated with * or ** for levels of 0.05 and 0.01, respectively.

The only SNP associated with natural variation in dry weight at 500mM NaCl (Fig. 3A) was located in the promoter region of At4g08850, encoding a member of the leucine-rich repeat receptor-like protein kinase family (Fig. 3B). This protein is predicted to have 24 LRR domains at its N-terminal extracellular domain (Val_44_–Leu_709_) followed by one helical transmembrane domain (Ile_710_–Ile_730_) and a cytosol-facing topological domain (Phe_731_–Ser_1045_) with a protein kinase domain localized at its C-terminus (Phe_775_–Ser_1045_). Natural variation in the promoter and gene coding sequence was examined for the sequenced accessions of the HapMap population and the sequence similarity, gaps and missing data were analysed (Fig. 3B). The promoter sequence upstream of the identified SNP as well as the sequence encoding the C-terminal protein kinase showed little conservation among the sequenced accessions. Since expression of At4g08850 was reported to be enhanced in the root tissue after exposure to salt stress (Fig. 3C, Kilian *et al.*, 2007), we named the gene *LRR-Kinase Induced by Salt Stress* (*LRR-KISS*). Natural variation in expression of *LRR-KISS* was observed previously (Lempe *et al.*, 2005), with Cen-0 showing enhanced, and HR-5 exhibiting reduced expression levels (Fig. 3D). These accessions showing contrasting levels of *LRR-KISS* expression were tested for salinity tolerance under mild salt stress conditions. One-week-old seedlings were watered with 75mM NaCl for 6 weeks in short day conditions after which the fresh weight of the rosette was determined. In control conditions, the rosettes of HR-5 were significantly larger than the rosettes of Col-0, while no significant difference was observed between Col-0 and Cen-0 (Fig. 3E). The rosettes of Cen-0 plants grown at salt stress conditions were significantly larger than the Col-0 rosettes. When the relative difference in the rosette size was examined, Cen-0 showed no alteration of rosette size by salt stress, while rosette growth of HR-5 was hypersensitive to salt stress (Fig. 3E).

**Fig. 4.**
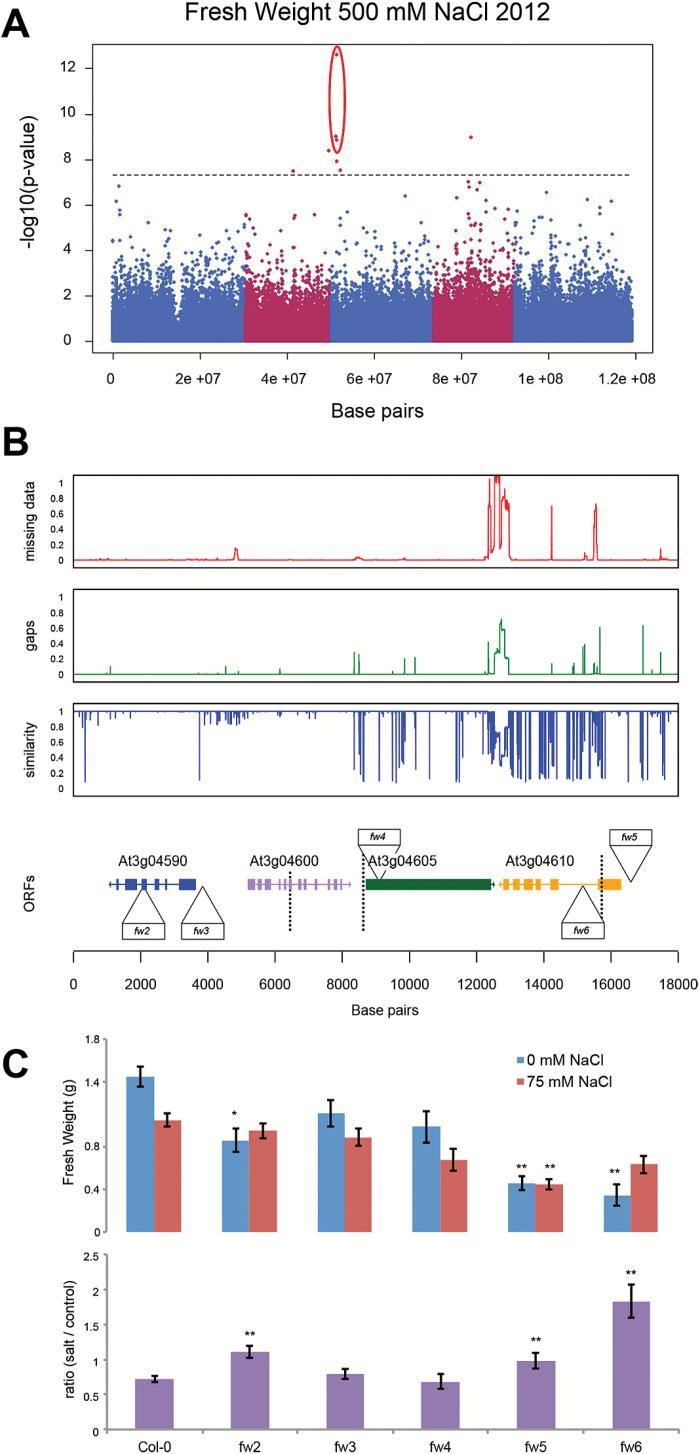
Natural variation in fresh weight associates with a cluster of genes containing transposable element MUSTANG1. (A) Genome wide association study performed on the fresh weight of above-ground tissue collected from plants grown at 500mM NaCl using minor allele frequency of 0.01 showed an association with 10 SNPs in seven distinct genomic loci (Table 3). The highest association was observed for an SNP on chromosome 3, which neighboured two other SNPs with significant LOD scores. The associations were not observed for any other trait or condition studied. (B) The genetic variation in the region was studied in 162 accessions sequenced by the 1001 Genomes Project belonging to the HapMap population (Supplementary Table S6). The red graph represents the portion of missing data, while the green graph represents deletions present in accessions other than Col-0. The blue graph represents the sequence similarity compared to Col-0. The open reading frames (ORFs) are aligned in the lowest graph. The location of the SNPs associated with FW at 500mM NaCl is represented by the dashed lines. The SNPs were around At3g04605-encoding transposable element MUSTANG1, but were also found in At3g04600, encoding nucleotidylyl transferase superfamily protein, and At3g04610, encoding flowering locus KH domain. Considering the linkage disequilibrium, putative candidate genes were extended to At3g04590, encoding AT hook motif DNA-binding family protein. The association was further confirmed by studying five available T-DNA insertion lines, whose location is indicated in the graph representing the ORFs. The SALK numbers and primers used for genotyping are listed in Supplementary Table S5. (C) The T-DNA insertion lines were tested for rosette growth in control and salt stress conditions. One-week-old plants grown under short-day conditions were treated with 0 or 75mM NaCl applied from above for 6 weeks. The fresh weight of the rosette was used as an indicator of growth and salinity tolerance. The bars represent the average fresh weight of the rosette as calculated from 15 biological replicates per line per condition. The error bars represent standard error. The relative values were calculated by dividing the rosette FW at salt stress by average FW in control conditions. The phenotypes of T-DNA insertion lines were tested for significant differences from Col-0, which were calculated using ANOVA with Tukey’s *post hoc* test. The significance is indicated with * or ** for levels of 0.05 and 0.01, respectively.

Fresh weight of above-ground tissue collected from plants grown at 500mM NaCl was found to be associated with six loci (Table 3), of which one locus was associated with three SNPs with highest LOD score 12.6 (Fig. 4A). The SNPs associated with this locus were clustered in one region on chromosome 3 spanning genes At3g04600 and At3g04610, with At3g04605 encoding a member of the transposable element gene family, MUSTANG1. Since the association in this region could be due to the presence or absence of the transposable element, the natural variation in the MUSTANG1 sequence and neighbouring genes was examined for the 162 sequenced accessions of the HapMap population (Fig. 4B). Although transposable element MUSTANG1 was present in all accessions, a number of sites with low sequence similarity were observed. The region spanning At3g04610, encoding the flowering locus KH domain protein, was observed to exhibit high allelic variation across the entire gene-coding region. To validate the role of the genes adjacent to the locus most significantly associated with fresh weight at 500mM NaCl, five T-DNA insertion lines were studied with regard to changes in salt stress tolerance (Fig. 4C). In control conditions, one T-DNA insertion line in At3g04590 (*fw2*) and two T-DNA insertion lines in At3g04610 (*fw5* and *fw6*) developed significantly smaller rosettes than reference line Col-0. Measuring the relative effect of salt stress on rosette size revealed that *fw2*, as well as *fw5* and *fw6*, was less affected by salt stress. Those results suggest that genes in the region associated with fresh weight of plants grown under severe salt stress might contribute to growth maintenance in salt stress conditions. However, scarce availability of genetic resources for the candidate genes prevents identification of the genes/alleles causal to the association identified with GWAS.

**Fig. 5.**
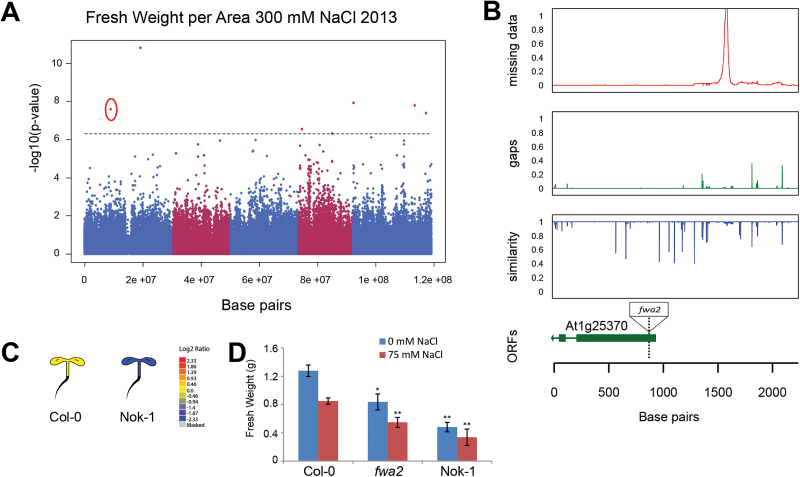
Natural variation in fresh weight per rosette area associates with unknown gene involved in rosette development. (A) Genome wide association study performed on the fresh weight per rosette area collected from plants grown at 300mM NaCl showed an association with five loci with low minor allele frequency above 0.02 (Table 3). One of the SNPs was located in the coding region of At1g25370, circled in red, encoding an unknown gene. The associations were not observed for any other trait or condition studied. (B) The natural variation in the region was studied in 162 accessions sequenced by the 1001 Genomes Project belonging to the HapMap population (Supplementary Table S6). The uppermost graph represents the portion of missing data, while the middle graph represents deletions present in accessions other than Col-0. The lowest graph represents the sequence similarity compared with Col-0. The open reading frames (ORFs) are aligned in the lowest graph. The location of the SNP associated with FW/PRA at 500mM NaCl is represented with the dashed line. The association was further confirmed by studying T-DNA insertion line, whose location is indicated in the graph representing the ORFs. The SALK number and primers used for genotyping are listed in Supplementary Table S5. (C) Natural variation in At1g25370 expression in previous studies revealed Nok-1 as a natural knockdown mutant (Lempe *et al.*, 2005) as reported by the eFP browser. Different colours represent the relative expression compared with Col-0. (D) The natural knockdown line Nok-1 and T-DNA insertion line *fwa2* were tested for rosette growth in control and salt stress conditions. One-week-old plants grown under short-day conditions were treated with 0 or 75mM NaCl applied from above for 6 weeks. The fresh weight of the rosette was used as an indicator of growth and salinity tolerance. The bars represent the average fresh weight of the rosette as calculated from 15 biological replicates per line per condition. The error bars represent standard error. The phenotypes of both lines were tested for significant differences from Col-0, which were calculated using ANOVA with Tukey’s *post hoc* test. The significance is indicated with * or ** for levels of 0.05 and 0.01, respectively.

No significant associations with fresh or dry weight were found for the plants treated with 300mM NaCl. Instead, the ratio between fresh weight and rosette area was used as a proxy for water retention in shoot tissue and therefore salt stress tolerance. Five loci with relatively low minor allele frequency (<0.02) were associated with this trait (Table 3). One of the associated SNPs, with a LOD score of 7.6, was located on chromosome 1 (Fig. 5A). The SNP was located in the first exon of At1g25370 encoding an unknown protein. The analysis of sequence similarity in the promoter and gene-coding region revealed little genetic diversity in the coding region and a number of polymorphisms in the promoter region (Fig. 5B). Natural variation in At1g25370 expression was observed previously (Lempe *et al.*, 2005), with Nok-1 showing reduced transcription (Fig. 5C). In order to examine the contribution of At1g25370 to salinity tolerance, this natural knockdown line, Nok-1, and a T-DNA insertion line (*fwa2*) (Fig. 5B) were examined for their rosette growth in control and salt stress conditions. Both *fwa2* and Nok-1 showed reduced rosette size in control and salt stress conditions (Fig. 5D). Those results suggest that low expression of unknown gene At1g25370 results in reduced rosette growth that also hinders plant growth under salt stress conditions.

## Discussion

In this study, we explored the effect of salinity on rosette size and natural variation therein, revealing a positive correlation between plant size and salt stress tolerance. In general we found a strong correlation between plant size in control and salt stress conditions (Table 1). Use of electrolyte leakage as a measure of cell damage (Bajji *et al.*, 2002) provided additional insight into plant conditions under salinity stress (Fig. 2A and Supplementary Fig S3E). Clustering of the accessions based on rosette size and electrolyte leakage revealed a clear and consistent pattern in which accessions with large rosettes exhibited less cell damage under salt stress conditions (Fig. 2B, C). This observation implies that larger plant volume dilutes sodium influx in the shoot tissue preventing sodium ion accumulation to concentrations at which sodium becomes toxic and plant growth is severely reduced.

No significant correlations were found for rosette size measured in our experiment, and ion accumulation data collected by Baxter *et al.* (2010) (Supplementary Tables S3 and S4). Although the experiments were conducted under different conditions, the lack of correlation would suggest that while ion homeostasis is a key factor, other mechanisms also contribute to plant growth under salt stress conditions. Similar trends were observed for some wheat varieties (Genc *et al.*, 2007) indicating that besides sodium exclusion, other mechanisms affecting tissue tolerance play an important role in salinity tolerance. The group of the accessions with large rosettes included accessions previously identified as salt tolerant, such as Tsu-0 and Bur-0 (Rus *et al.*, 2006; Katori *et al.*, 2010). Weaker correlations between rosette development in control and severe salt stress (500mM NaCl, Table 1) imply that mechanisms other than growth maintenance, for instance ion exclusion, play a more dominant role in severe salt stress conditions. Our results show the value of assessing the salinity tolerance based on the rosette development on multiple levels, such as projected rosette area, weight and electrolyte leakage.

Two accessions, An-1 and Pa-2, were identified as the outliers to the general trend, showing low cell damage despite their small size (Fig. 2C). It is very likely that the mechanisms underlying salinity tolerance in those accessions are different from rosette development and could include efficient ion exclusion from the transpiration stream or enhanced tissue tolerance due to ion compartmentalization into the vacuoles. Therefore, An-1 and Pa-2 are interesting candidate accessions for further investigation of the molecular and genetic basis of their salinity tolerance. However, as those types of accessions are scarce, the allele frequency of genes underlying salinity tolerance in slow growing accessions is likely low and therefore they are more suitable for investigation in recombinant inbred lines population QTL studies (Kover and Mott, 2012).

GWAS revealed a number of candidate genes underlying rosette development under control and salt stress conditions. Although significant correlations were observed between the rosette size in control and salt stress conditions, no overlapping associations were shared between salt and control conditions, indicating that genetic variation responsible for rosette development differs between control and salt stress conditions. None of the candidate genes underlying the associations was previously described as being involved in salt stress tolerance. Since GWAS is known to be prone to false positive associations, the candidate loci remain tentative until further validation is provided. In order to confirm a selected number of associations, available T-DNA lines and accessions previously established to have altered transcription levels of candidate genes (Lempe *et al.*, 2005) were studied in more detail for their rosette phenotypes. While in the large scale experiments (Supplementary Fig. S1) plants were watered with high salt from the bottom, the individual mutant lines were treated at an earlier developmental stage with low salt applied from above, which is thought to better reflect saline conditions in the field to reveal potential adaptive gain of candidate genes for salinity tolerance. Additionally, to pinpoint the genetic region causal to association, local genetic variation was examined by comparing the genetic regions proximal to associated SNPs of the sequenced accessions (Ossowski *et al.*, 2008; Gan *et al.*, 2011) and identification of the regions rich in polymorphisms.

Both approaches were complementary in the case of the candidate gene identified with dry weight at 500mM NaCl, *LRR-KISS* (Fig. 3), which showed enhanced expression in salt stress conditions (Kilian *et al.*, 2007). The domain encoding the kinase domain was observed to contain multiple SNPs in several accessions (Fig. 3B), which might affect its kinase activity. Our results suggest that high *LRR-KISS* expression would enhance rosette growth maintenance under salt stress conditions (Fig. 3D, E); however the alleles causal to high *LRR-KISS* expression remain to be identified. For two other identified associations, corresponding to a KH domain-containing protein and an unknown protein, analysis of T-DNA insertion lines and accessions carrying a low-expression allele suggested that those associations are linked to rosette development rather than specific response to salinity. Interestingly, since rosette development is tightly linked to salinity tolerance, the allelic variation in those candidate genes may still contribute to adaptation to saline environments.

Our results illustrate an important aspect of abiotic stress biology, which is that salinity tolerance is linked to growth and development under control conditions. Low-cost phenotyping methods estimating the rosette biomass, area and electrolyte leakage allowed identification of novel putative candidate genes in Arabidopsis rosette development and salt stress tolerance. Our preliminary validation of the candidate genes identified by means of GWAS confirms their involvement in rosette development as well as salt stress-specific inhibition of rosette growth. Further characterization of candidate genes identified will provide a better insight in the processes involved in shoot growth maintenance under salinity stress and the major molecular players therein.

## Supplemental data

Supplementary data are available at *JXB* online. 

Figure S1. Assessment of salinity tolerance in Arabidopsis accessions. Experimental set-up.

Figure S2. The correlation between two experiments performed.

Figure S3. Natural variation in all rosette size-related phenotypes studied.

Figure S4. The correlation between different rosette phenotypes collected from plants grown under salt stress conditions.

Table S1. Accessions used for the experiment performed in 2012.

Table S2. Accessions used for the experiment performed in 2013.

Table S3. Correlations between rosette phenotypes collected in the experiment performed in 2012 and ion accumulation collected by Baxter *et al.* (2010).

Table S4. Correlations between rosette phenotypes and electrolyte leakage collected in the experiment performed in 2013 and ion accumulation collected by Baxter *et al.* (2010).

Table S5. T-DNA insertion lines and primers for genotyping.

Table S6. Accession sequences used for the study of sequence similarity retrieved from the 1001 Genomes Project website (1001genomes.org).

## Supplementary Material

Supplementary Data
