# Supplementary material for: Natural variation in rosette size under salt stress conditions corresponds to developmental differences between Arabidopsis accessions and allelic variation in the LRR-KISS gene
Source: J Exp Bot. 2016 Feb 11;67(8):2127–38. doi: 10.1093/jxb/erw015 (PMC4809279; doi:10.1093/jxb/erw015)
Supplement: Supplementary Data [file supp_67_8_2127__index.html]

Natural variation in rosette size under salt stress conditions corresponds to developmental differences between Arabidopsis accessions and allelic variation in the LRR-KISS gene — Supplementary Data 

# Natural variation in rosette size under salt stress conditions corresponds to developmental differences between Arabidopsis accessions and allelic variation in the *LRR-KISS* gene

## Supplementary Data

Data files

- supplementary\_figures\_S1\_S4.pdf - Supplementary Data
- supplementary\_tables\_S1\_S6.xlsx - Supplementary Data
- batch\_final.doc - Supplementary Data
