# Supplementary material for: Natural variation in rosette size under salt stress conditions corresponds to developmental differences between Arabidopsis accessions and allelic variation in the LRR-KISS gene
Source: J Exp Bot. 2016 Feb 11;67(8):2127–38. doi: 10.1093/jxb/erw015 (PMC4809279; doi:10.1093/jxb/erw015)
Supplement: Supplementary Data [file supp_erw015_supplementary_figures_S1_S4.pdf]

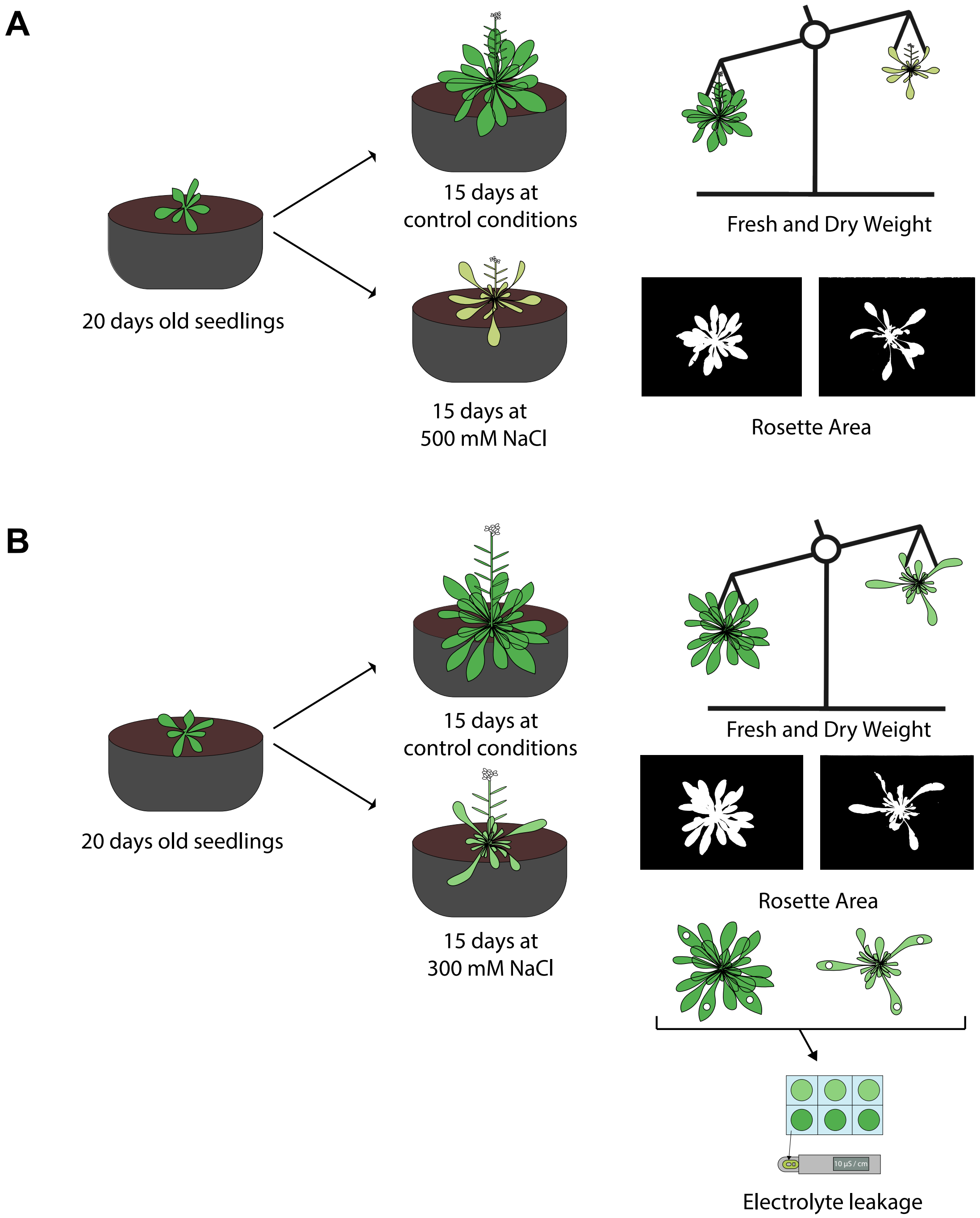

Figure S1. Assessment of salinity tolerance in Arabidopsis accessions. Experimental set up. Two populations of Arabidopsis accessions were used in two independent experiments performed in greenhouse conditions in years 2012 and 2013. The accessions used in two experiments are listed in Table S1 and S2 respectively. **(A)** Twenty days old plants were watered with 0 or 500 mM NaCl for 15 days after which the fresh and dry weight of the rosette with flowering stems were measured. The pictures of the rosettes were taken from above and the projected rosette area was calculated in ImageJ. Four replicates per accession were measured. **(B)** Twenty days old plants were watered with 0 or 300 mM NaCl for 15 days after which the fresh and dry weight of the rosette was measured. The pictures of the rosettes were taken from above and the projected rosette area was calculated in ImageJ. Three leaf discs per plant were taken for electrolyte leakage measurements. Three replicates

per accession per condition were used for dry weight and electrolyte leakage measurement. Fresh weight and projected rosette area were collected from six biological replicates.

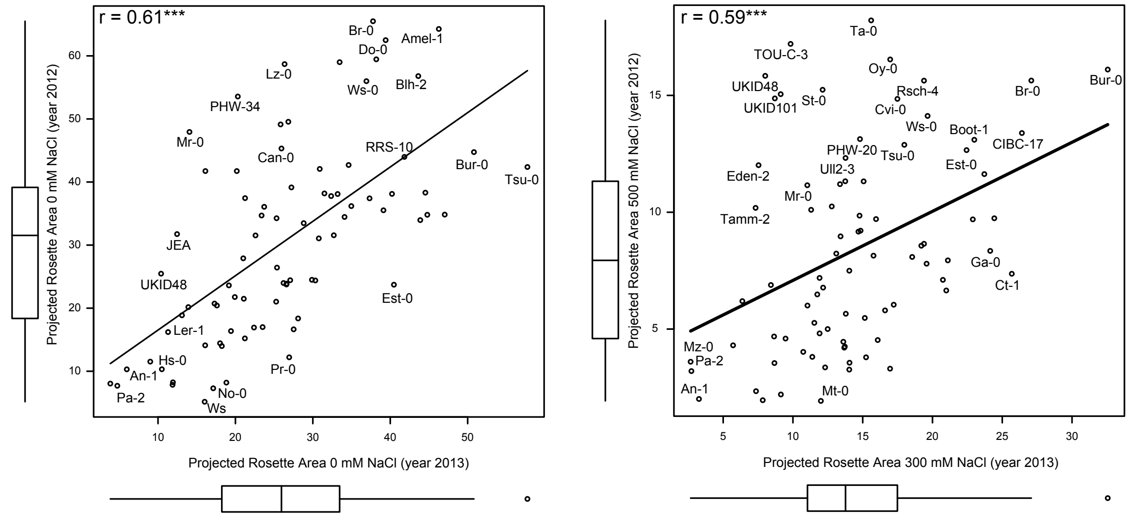

Figure S2. The correlation between two experiments performed. The rosette size related phenotypes were scored for 35 days old plants treated with 0 or 300 /500 mM NaCl for 15 days. The phenotypes of 81 overlapping accessions between two experiments were used for calculating Pearson correlation coefficients (r) between the experiments. The Projected Rosette Area in control and salt stress conditions was significantly correlated between both experiments. The scatter plots represent the average phenotype observed for individual accessions calculated from 4 biological replicates in experiment performed in 2012 and 6 biological replicates in experiment performed in 2013. Pearson correlation coefficient values (*r*) are listed in upper left corner of each scatter plot. The significance is indicated with * or ** for significance levels of 0.05 and 0.01 respectively.

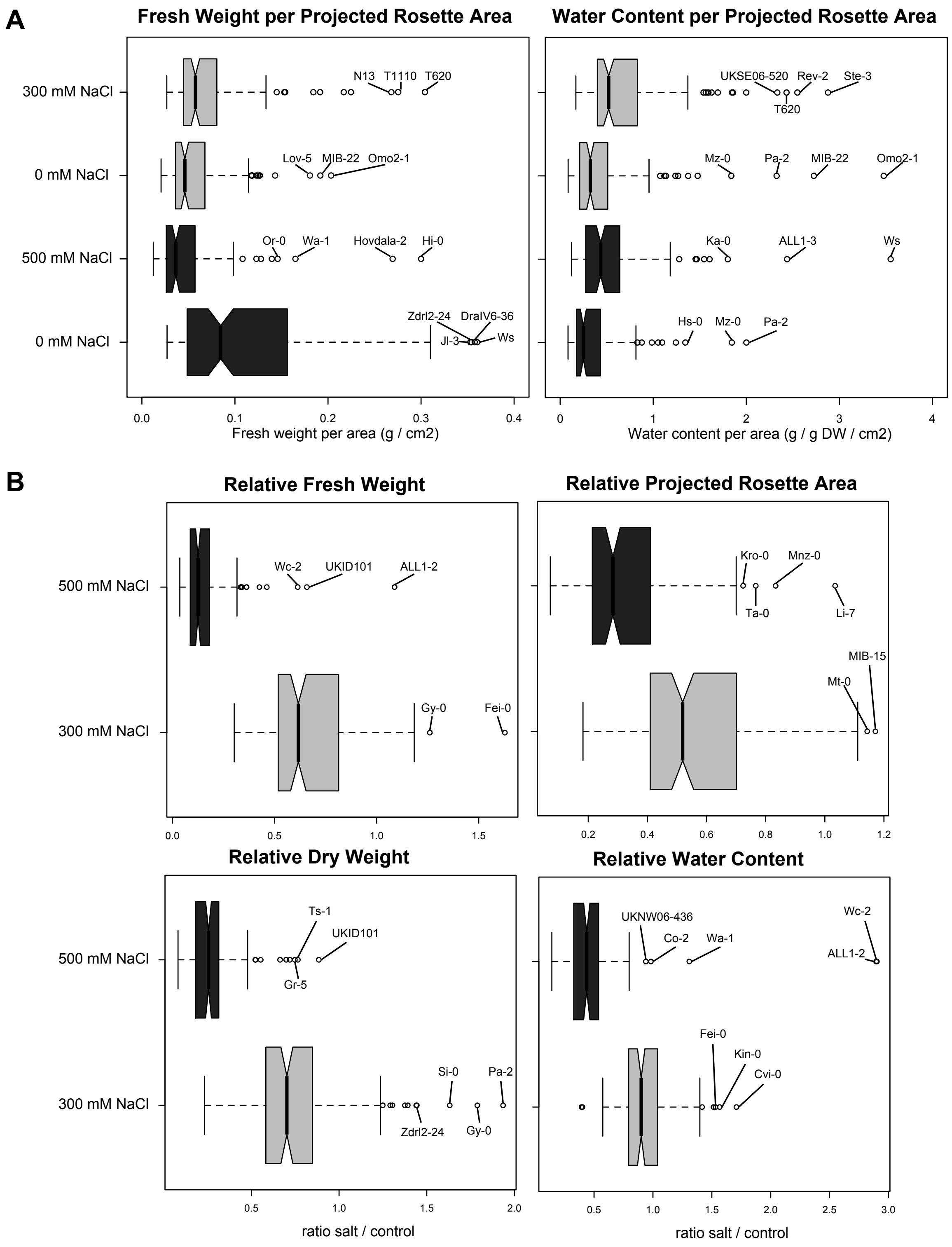

Figure S3. Natural variation in all rosette size related phenotypes studied. Natural variation in rosette parameters was observed for two populations consisting of 160 accessions each in two separate experiments performed in 2012 and 2013 using 300 and 500 mM NaCl as salt stress treatment respectively. Several accessions were determined as outliers in each rosette size related parameter studied. **(A)** The boxplots represent the median of 160 accessions of Fresh Weight per Rosette Area (left panel) and Water

Content per Projected Rosette Area (dark panel). Fresh Weight and Dry weight of rosette and flowering stem was measured in experiment conducted in year 2012 (dark box plots), while in experiment conducted in 2013 Fresh and Dry weight of rosette was measured. **(B)** The relative effect of salt stress on rosette growth was determined for both experiments by dividing the average phenotype value at salt stress conditions by the average value measured at control conditions for Fresh Weight, Dry Weight, Projected Rosette Area and Water Content. The boxplots represent the median values as measured in the populations consisting of 160 accessions and 113 accessions for Electrolyte leakage. The whiskers extend to data points that are less than 1.5x from interquartile range (IQR) away from 1st and 3rd quartile. Notches represent 1.58 x IQR / sqrt (n) and give 95% confidence that two medians differ. Accessions representing outliers for a trait are indicated.

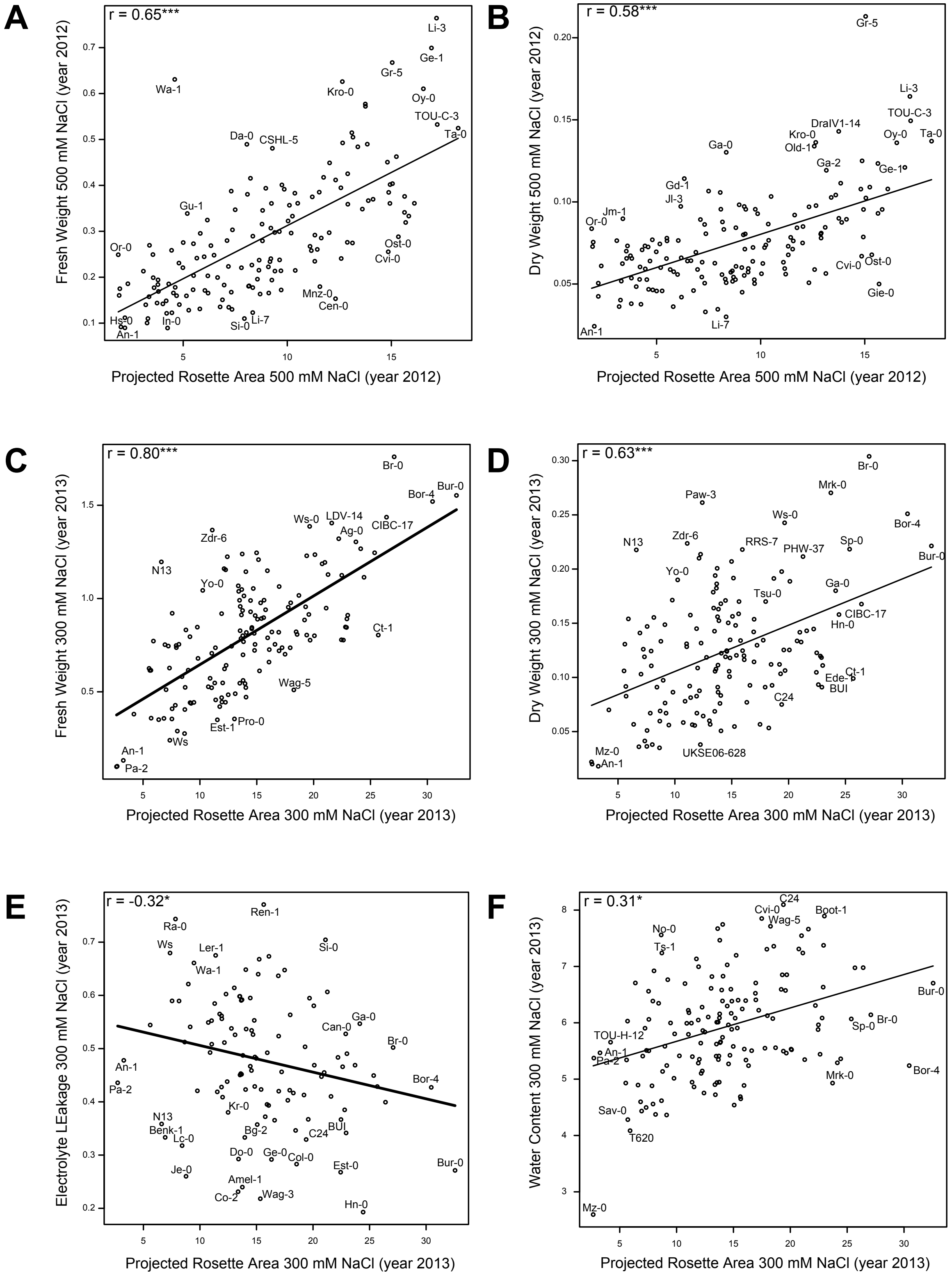

Figure S4. The correlation between different rosette phenotypes collected from plants grown under salt stress conditions. The rosette size related phenotypes were scored for 35 days old plants treated with 300 or 500 mM NaCl for 15 days. In the experiment performed in 2012 fresh and dry weight were determined for the rosette and flowering stem, while in experiment performed in year 2013 fresh and

dry weight were determined for the rosette only. The phenotypes of 160 accessions were used for calculating Pearson correlation coefficients (r) per experiment. In the experiment performed in 2012 **(A)** the correlation between Projected Rosette Area and Fresh Weight and **(B)** between Projected Rosette Area and Dry Weight was observed to be significant. In the experiment performed in 2013, significant correlations were observed between **(C)** Projected Rosette Area and Fresh Weight, **(D)** Projected Rosette Area and Dry Weight **(E)** Projected Rosette Area and Electrolyte Leakage and **(F)** Projected Rosette Area and Water Content. The scatter plots represent the average phenotype observed for individual accessions calculated from 4 biological replicates in experiment performed in 2012 and 6 biological replicates for FW and PRA and 3 biological replicates for DW in experiment performed in 2013. Pearson correlation coefficient values (*r*) are listed in upper left corner of each scatter plot. The significance is indicated with * or ** for significance levels of 0.05 and 0.01 respectively.
