# Supplementary material for: Natural variation in rosette size under salt stress conditions corresponds to developmental differences between Arabidopsis accessions and allelic variation in the LRR-KISS gene
Source: J Exp Bot. 2016 Feb 11;67(8):2127–38. doi: 10.1093/jxb/erw015 (PMC4809279; doi:10.1093/jxb/erw015)
Supplement: Supplementary Data [file supp_erw015_batch_final.doc]

run("Set Scale...", "distance=80.444 known=1 pixel=1 unit=cm");

run("Set Measurements...", "area area_fraction display redirect=None decimal=3");

run("Color Threshold...");

// Color Thresholder 1.44o

// Autogenerated macro, single images only!

min=newArray(3);

max=newArray(3);

filter=newArray(3);

a=getTitle();

run("HSB Stack");

run("Convert Stack to Images");

selectWindow("Hue");

rename("0");

selectWindow("Saturation");

rename("1");

selectWindow("Brightness");

rename("2");

min[0]=3;

max[0]=255;

filter[0]="pass";

min[1]=65;

max[1]=255;

filter[1]="pass";

min[2]=0;

max[2]=216;

filter[2]="pass";

for (i=0;i<3;i++){

selectWindow(""+i);

setThreshold(min[i], max[i]);

run("Convert to Mask");

if (filter[i]=="stop") run("Invert");

}

imageCalculator("AND create", "0","1");

imageCalculator("AND create", "Result of 0","2");

for (i=0;i<3;i++){

selectWindow(""+i);

close();

}

selectWindow("Result of 0");

close();

selectWindow("Result of Result of 0");

rename(a);

// Colour Thresholding-------------

run("Measure");

close();
